# Acute pain and analgesic prescribing in trauma inpatients at a tertiary hospital: A cross-sectional study

**DOI:** 10.4102/jcmsa.v4i1.315

**Published:** 2026-08-19

**Authors:** Sarah A. van der Spuy, Prashil Daya, Saxon Leathwhite, Zanele Mangena, Thato Motaung, Mikaela G. Naidoo, Anzelle Pretorius, Thifhelimbilu E. Luvhengo

**Affiliations:** 1 Unit of Undergraduate Medical Education, School of Clinical Medicine, Faculty of Health Sciences, University of the Witwatersrand, Johannesburg, South Africa; 2 Department of Surgery, School of Clinical Medicine, Faculty of Health Sciences, University of the Witwatersrand, Johannesburg, South Africa

**Keywords:** acute pain, trauma, analgesic prescribing, cross-sectional study, South Africa

## Abstract

**Background:**

Acute pain after trauma remains common and is often not well controlled. This study described pain intensity at inpatient assessment and the analgesics prescribed to adults admitted after traumatic injury to a tertiary academic trauma unit.

**Methods:**

This was a cross-sectional observational study of adult trauma patients admitted to the general trauma ward. Data collected included demographics, comorbidities, current pain intensity on the Numerical Rating Scale, psychological symptom scores, substance-use risk and prescribed analgesics. Reported pain severity at admission was ascertained from the patient. Categorical data were summarised descriptively, and exploratory associations were assessed using chi-squared or Fisher’s exact test, as appropriate.

**Results:**

The study included 143 participants, of whom 81.1% (*n* = 116) were male, and 33.6% (*n* = 48) had penetrating injury. At interview, 62.9% (*n* = 90) reported moderate to severe pain. Paracetamol with tramadol was the most commonly prescribed regimen across pain categories. No statistically significant association was observed between current pain category and hypertension, diabetes mellitus, human immunodeficiency virus status, anxiety symptoms, depressive symptoms, or substance-use risk.

**Conclusion:**

Most participants were still experiencing moderate to severe pain at the time of assessment despite prescribed analgesia. Paracetamol with tramadol remained the dominant regimen, with limited visible change across pain categories.

**Contribution:**

This study provides an inpatient, cross-sectional account of persistent pain after trauma and the analgesics prescribed in a South African tertiary trauma service and may inform local quality-improvement efforts in trauma pain management.

## Introduction

The International Association for the Study of Pain (IASP) defines pain as an ‘unpleasant sensory and emotional experience associated with, or resembling that associated with, actual or potential tissue damage’.^1^ Severe acute pain is common after trauma and critical illness, with studies reporting moderate to severe pain in many patients despite treatment.^2^ Inadequately controlled acute pain may prolong hospitalisation, impair quality of life, and contribute to poorer clinical outcomes.^2,3^ Biological consequences of poorly controlled acute pain include activation of the hypothalamic-pituitary-adrenal axis, sympathetic stress responses, immunological dysfunction and aggravation of comorbid conditions.^4^

Several patient factors, including sex, age, mental health symptoms, human immunodeficiency virus (HIV) infection, hypertension, diabetes mellitus, and substance use, may shape the pain experience and analgesic requirements after injury.^5,6,7,8,9,10^ These factors are clinically relevant because they may influence how pain is reported, how clinicians prescribe analgesia and how difficult pain is to control in individual patients.

Structured approaches to acute trauma pain management emphasise multimodal pharmacological therapy, repeated reassessment, opioid stewardship and regional techniques where appropriate.^11,12,13^ The World Health Organization (WHO) analgesic ladder remains a useful historical and potency-based framework, but it was developed for cancer pain and does not fully capture contemporary trauma pathways.^14,15^

Recent South African evidence from the Western Cape prehospital setting and from a Cape Town emergency-centre audit shows that pain assessment and analgesic delivery remain inconsistent across the trauma-care continuum.^16,17^ Against that background, the present study adds an inpatient perspective from a tertiary hospital and links ongoing pain during admission to the analgesics prescribed in routine practice, rather than focusing solely on triage or emergency-centre care.

Pain should be recognised early and quantified, but trauma pain remains difficult to characterise because intensity is influenced by tissue injury, psychological distress, prior substance exposure and individual variability. Simple unidimensional tools such as the Numerical Rating Scale (NRS) remain practical for routine clinical use, especially in resource-constrained settings.^18,19^ The Patient Health Questionnaire (PHG-9) and the Generalised Anxiety Disorder 7 scale (GAD-7) are not pain scales; rather, they are validated screening tools for depressive and anxiety symptoms that may contextualise how pain is experienced and reported after injury.^20,21^

The Drug Abuse Screening Test-10 (DAST-10) can help quantify substance-use risk in patients in whom prior substance exposure may complicate acute pain management.^10^ In this study, the NRS was used to categorise current pain intensity (score) as no (0), mild (1–3), moderate (4–6), severe (7–9), and worst imaginable (10). The study also recorded reported pain severity on admission where available in order to describe prescribing patterns in relation to initial pain severity.

Understanding current pain intensity and prescribing practice in trauma inpatients is important if modifiable gaps in care are to be identified, particularly in resource-constrained tertiary services where default prescribing habits may become entrenched. This study therefore aimed to describe pain intensity at the time of interview in patients admitted to a Level 1 Trauma Unit of public hospital in South Africa, to describe the analgesic regimens prescribed and to explore whether selected comorbid and psychosocial factors were associated with pain intensity at the time of interview. Reporting in this manuscript is according to the Strengthening the reporting of cohort, cross-sectional and case-control studies in surgery (STROCSS) Checklist.^22^

## Research methods and design

This was a cross-sectional observational quantitative study of adults admitted following traumatic injury to a Level 1 Trauma Unit of a public hospital in South Africa. The unit receives patients directly from the scene or as referrals from other hospitals. The estimated sample size was 387.

Participants were 18 years or older with a Glasgow Coma Scale (GCS) of 15/15. A pilot study was conducted before commencement of the project. The study included patients who presented or were brought to the hospital directly from the scene of injury and excluded transfers. Also excluded were patients with prior analgesic use within the past week and those who were part of the pilot study. We used the convenience sampling method.

Data were obtained through a standardised interview and review of medical records. We captured data onto the Research Electronic Data Capture (REDCap) platform. Current pain intensity was recorded at interview using the NRS. Where documented, reported pain severity on admission was abstracted from the patient chart to describe prescribing patterns in relation to initial pain severity. Admission pain category was missing for eight participants, and these participants were therefore excluded from analyses.

Data were exported from REDCap and analysed using Excel and Stata. Numerical Rating Scale scores were categorised as no (0), mild (1–3), moderate (4–6), severe (7–9) or worst pain imaginable, as described previously (10). For descriptive purposes, an NRS score of 4 or greater was considered clinically significant pain, consistent with prior acute pain literature.^19^ Analgesics were grouped descriptively according to regimen and opioid potency.

Exploratory associations between pain category at the time of interview and selected demographic, comorbid and psychosocial variables were compared using the chi-squared or Fisher’s test, as appropriate. The level of significance was set a *p*-value of < 0.05. Because the achieved sample was substantially smaller than planned, particularly within several comorbidity and psychosocial subgroups, these analyses were interpreted cautiously and regarded as exploratory rather than definitive.

### Ethical considerations

Ethical clearance to conduct this study was obtained from the University of the Witwatersrand, Human Research Ethics Committee (No. M231036/B/0001). Ethical approval was received and written informed consent obtained from the participants before commencement of the study. We ensured confidentiality by assigning a unique study code for each participant and storing data in a secure database.

## Results

**FIGURE 1:**
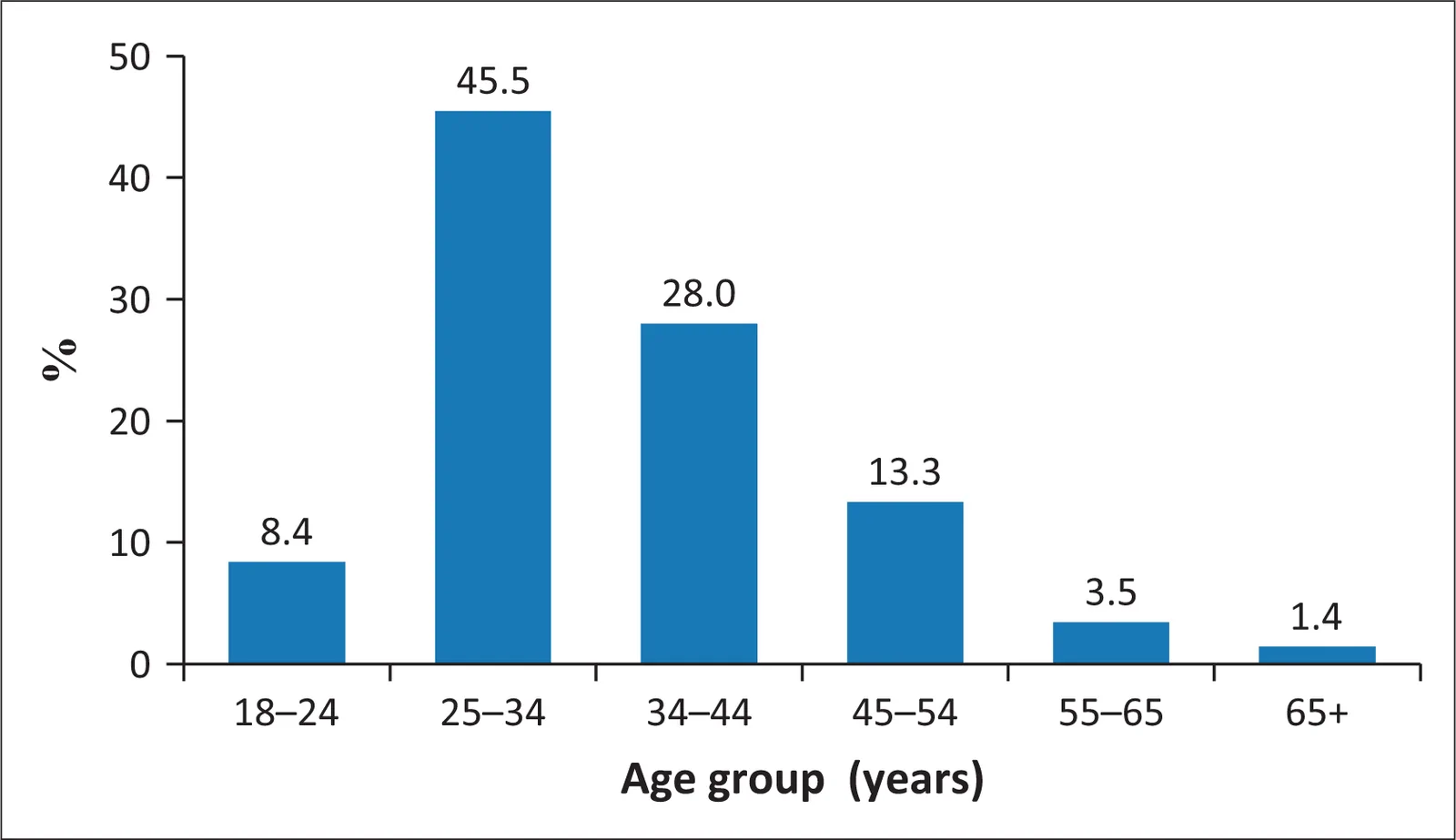
Age distribution of the participants (*N* = 143).

A total of143 participants were included, of whom 81.1% (*n* = 116) were male, and 18.9% (*n* = 27) were female. The age distribution was concentrated in the 25–34-year and 35–44-year groups, comprising 45.5 and 28% of the sample, respectively (Figure 1).

**FIGURE 2:**
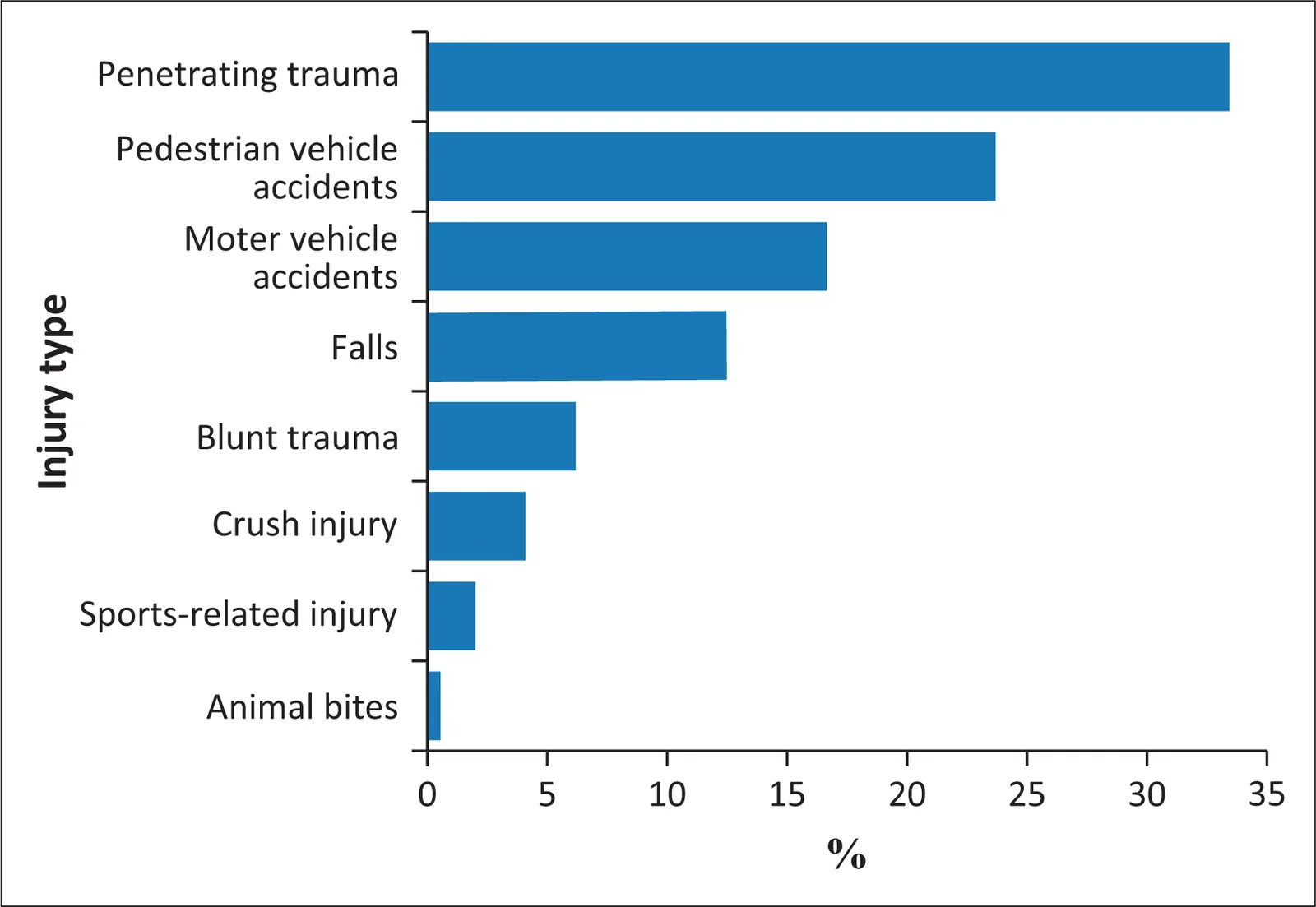
Breakdown of mechanisms of injury among participants (*N* = 143).

Mechanisms of injury included penetrating trauma in 33.6% and pedestrian-vehicle accidents in 23.8% of participants (Figure 2).

**FIGURE 3:**
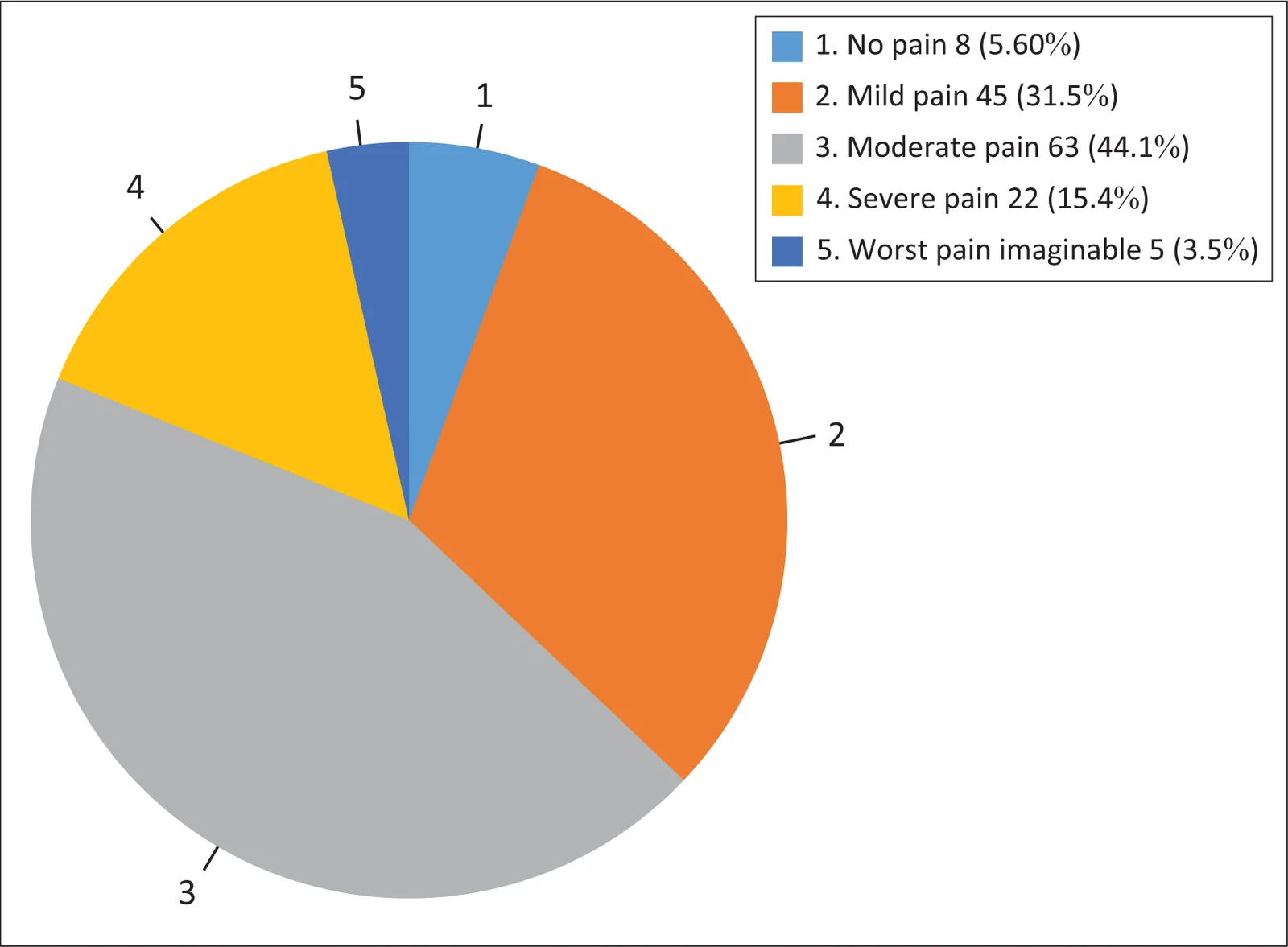
Reported pain intensity levels at the time of interview during admission (*N* = 143).

At the time of interview, 62.9% of participants reported moderate to severe pain (NRS 4–10). Mild pain was reported by 31.5%, while 5.6% reported no pain at the time of assessment (Figure 3).

Comorbidities were infrequent in this cohort, with diabetes mellitus and hypertension each present in 3.5% of participants, while 21% tested positive for HIV.

In exploratory analyses, no statistically significant association was observed between current pain category and hypertension (*p* = 0.158), diabetes mellitus (*p* = 0.836) or HIV status (*p* = 1.000). Given the small numbers within several comorbidity groups, these findings should be interpreted cautiously. Moderate to severe anxiety symptoms were present in 11.9% of participants, moderate to severe depressive symptoms in 10.5% and 21.7% disclosed moderate to severe substance-use concern (Table 1).

**TABLE 1:** GAD-7 anxiety and PHQ-9 depression symptoms and substance-use risk levels among participants (*N* = 143).

Risk level	*n*	%
**GAD-7 anxiety symptom risk level and scores**		
No or minimal anxiety symptoms (Score: 0–4)	102	71.3
Mild anxiety symptoms (Score: 5–9)	24	16.8
Moderate anxiety symptoms (Score: 10–14)	11	7.7
Severe anxiety symptoms (Score: 15–21)	6	4.2
**PHQ-9 depressive symptom risk level and scores**		
No or minimal symptoms (Score: 0–4)	111	77.6
Mild symptoms (Score: 5–9)	17	11.9
Moderate symptoms (Score: 10–14)	10	7
Moderately severe symptoms (Score: 15–19)	4	2.6
Severe symptoms (Score: 20–27)	1	0.7
**DAST-10 substance-use risk level**		
No risk (Score: 0)	104	72.7
Low risk (Score: 1–2)	8	5.6
Moderate risk (Score: 3–5)	22	15.4
Substantial risk (Score: 6–8)	8	5.6
Severe risk (Score: 9–10)	1	0.7

DAST, drug abuse screening test; GAD, generalised anxiety disorder; PHQ, patient health questionnaire.

Among the 17 participants with moderate to severe anxiety symptoms (GAD-7 score ≥ 10), most reported moderate pain (65%). An exploratory association between anxiety symptom severity and current pain category did not reach statistical significance (Fisher’s exact *p* = 0.124) (Table 2).

**TABLE 2:** Levels of pain reported by participants with moderate or severe anxiety and depressive symptoms and moderate to severe risk of substance use.

Group	No pain		Mild pain		Moderate pain		Severe pain	
	*n*	%	*n*	%	*n*	%	*n*	%
Moderate or severe anxiety symptoms (*n* = 17)	1	6	3	18	11	65	2	12
Moderate or severe depression symptoms (*n* = 15)	1	7	5	33	8	53	1	7
Moderate or severe substance-use risk (*n* = 31)	0	0	14	45	16	52	1	3

Majority, 53.3% of participants with moderate to severe depressive symptoms (PHQ-9 score ≥ 10; *n* = 15), reported moderate pain (53%), followed by mild pain (33%). The test of association between depressive symptom severity and the pain severity did not yield statistically significant result (*p* = 0.168) (Table 2).

Of the participants with moderate to severe risk of substance use (DAST-10 score ≥ 3; *n* = 31), most reported moderate pain (51.6%), followed by mild pain (45.2%). However, the association between substance-use risk and current pain category did not reach statistical significance (*p* = 0.408) as shown in Table 2.

Table 3 lists the analgesics that were used across all pain intensities.

**TABLE 3:** Analgesics prescribed abstracted from participants’ prescription charts and severity of pain (*N* = 135; missing for eight participants).

Pain severity (on admission)	Analgesia used	Percentage used
Mild pain (1–3)	Tramadol + Pethidine	50.0
	Paracetamol + Tramadol	50.0
Moderate pain (4–6)	Pethidine + NSAIDs	12.5
	Paracetamol + Pethidine	12.5
	Paracetamol + Tramadol + Morphine	12.5
	Paracetamol + Tramadol + Pethidine	12.5
	Paracetamol + Tramadol	50.0
Severe pain (7–9)	Pethidine + NSAIDs	2.4
	Tramadol + Codeine	2.4
	Paracetamol + NSAIDs	2.4
	Paracetamol	2.4
	Paracetamol + Pethidine	2.4
	Paracetamol + Tramadol + Pethidine	9.5
	Paracetamol + Tramadol	78.6
Worst pain imaginable (10)	Pethidine	1.2
	Tramadol + NSAIDs	1.2
	Paracetamol + Morphine	1.2
	Paracetamol + Pethidine	1.2
	Paracetamol + Tramadol + NSAIDs	2.4
	Paracetamol	3.6
	Tramadol	4.8
	Paracetamol + Tramadol + Morphine	4.8
	Paracetamol + Tramadol + Pethidine	9.6
	Paracetamol + Tramadol	69.9

Note: The use of pethidine either alone or in combination with another opioid such as tramadol is discouraged.

NSAIDs, non-steroidal anti-inflammatory drugs.

Based on reported pain severity on admission abstracted from the patient chart, the dominant prescribing approach was a non-opioid plus weak-opioid regimen, most often paracetamol with tramadol, across pain categories. Although the use of pethidine is discouraged, it was prescribed in some cases. Worrisomely, in some patients pethidine was combined with tramadol (Table 3). In patients with moderate pain on admission, paracetamol with tramadol accounted for half of the regimens recorded. Among those with severe pain, the same combination remained the most common regimen, while strong-opioid-containing regimens were used less often. Admission pain category data were missing for eight participants and were not included in this analysis.

## Discussion

This cross-sectional study examined pain intensity and analgesic prescribing among adult trauma patients admitted to a tertiary academic hospital. The clearest finding was that 63% of participants still had moderate to severe pain despite prescribed analgesia. The prescribing data also showed that paracetamol with tramadol remained the commonest regimen across pain categories, with little sign of a stepwise change, as reported, pain severity increased. This supports concern that, even after admission, a substantial proportion of trauma patients continue to experience clinically important pain despite ongoing treatment.

Most participants were young adult males. This is broadly consistent with South African trauma populations, in which young men account for a large proportion of presentations to urban trauma services.^23,24^ The pattern likely reflects the concentration of trauma risk within the economically active population and among individuals with greater exposure to transport-related injury and interpersonal violence.

The profile of our cohort is also consistent with broader urban South African trauma epidemiology. Recent local data show that trauma in public-sector centres disproportionately affects young adult males and is driven largely by interpersonal violence and road-traffic injury, with penetrating trauma remaining common and pedestrian-related injury contributing substantially to the workload of tertiary services.^24^ This context is important when interpreting the present findings, because pain management in such units takes place within a high-volume environment shaped by service pressure, trauma severity and resource constraints.

Comorbid and psychosocial variables were examined because they may affect pain reporting, analgesic requirements and clinical complexity. However, the study found no statistically significant association between pain category at interview and the measured comorbid or psychosocial variables. Given the small subgroup counts and the shortfall from the planned sample size, these findings should be interpreted as inconclusive rather than reassuring.

The absence of a statistically significant association in this study should not be interpreted as evidence that anxiety and depressive symptoms are unimportant in trauma-related pain. Other injury studies have shown that anxiety, depression and broader post-injury psychological distress are associated with worse pain trajectories and poorer recovery outcomes over time, including reduced health and well-being quality of life, higher healthcare utilisation and delayed return to normal daily activities.^25,26^ In the present cohort, the lack of a clear association is more appropriately interpreted as inconclusive than negative, given the small number of participants with higher symptom scores, the cross-sectional design and the limitations of brief screening tools in an acutely injured inpatient population. These findings therefore support further prospective work examining pain and psychological symptoms over time, rather than dismissal of their clinical relevance.

The practical finding was how narrow the prescribing profile appeared to be. Although the WHO analgesic ladder remains a familiar historical and potency-based framework, it was developed for cancer pain and does not fully reflect current trauma pain management.^14,15^ Contemporary trauma guidance places more emphasis on multimodal analgesia, opioid stewardship, repeated reassessment and timely use of regional techniques where feasible.^11,12,13^. Against that standard, the repeated use of paracetamol with tramadol across all pain categories is noteworthy, especially when the same combination was often recorded even in patients who reported severe or worst pain on admission. This does not, in itself, prove inappropriate prescribing in individual cases, because the clinical rationale, contraindications, haemodynamic status and medication availability were not assessed; however, it does suggest limited observable variation in analgesic escalation within the cohort. Evidence syntheses in acute musculoskeletal injury also suggest limited benefit from tramadol, with greater harms across opioid strategies than with non-steroidal anti-inflammatory drug (NSAID)-based approaches, which supports closer scrutiny of routine weak-opioid default prescribing.^27^

Taken together, the findings point more towards a problem with the escalation of analgesic care than to any single patient-level factor. This study did not examine clinician prescribing rationale, actual drug administration, stock availability or access to regional and other non-pharmacological approaches, so it cannot explain why stronger escalation was uncommon. Regional analgesia was not specifically captured in the dataset. That omission matters because current trauma pathways increasingly include site-specific blocks and other multimodal strategies where local capacity permits.^28,29,30^

In practical terms, these findings support routine pain scoring at admission, reassessment after analgesia, clearer escalation pathways and protocolised multimodal order sets rather than complete reliance on individual prescriber preference.^11,12,13^ In resource-constrained services, these steps may be more achievable than immediate expansion to highly specialised techniques while still creating a clearer route for anaesthesia or regional analgesia referral where feasible.^16,30^ Further work should ideally combine serial pain assessment with better documentation of prescribed and administered analgesia, so that treatment response can be evaluated more directly and barriers to escalation can be identified more clearly.

### Limitations

The limitations of the study include that the planned sample size was 387, but only 143 participants were enrolled. This under-recruitment, together with the low prevalence of several comorbidities, reduced statistical power, especially for subgroup analyses. Additionally, the study was carried out at one tertiary academic hospital and used convenience sampling, which limit generalisability. Age and pain were analysed primarily in categories for reporting purposes, reducing granularity. Language barriers during some interviews may also have affected the precision of symptom reporting.

The analysis was cross-sectional and focused on pain intensity at one point during admission. It therefore cannot determine change in pain over time or establish the effectiveness of analgesic therapy. In addition, the analysis focused on prescribed pharmacological agents and did not evaluate actual administration, dosing timing, clinician rationale or non-pharmacological and regional analgesic techniques. PHQ-9 and GAD-7 were used as contextual psychological screening tools, but their interpretation in an acute trauma inpatient population has limitations because of time frame assumptions and symptom overlap. These limitations mean that the subgroup findings should be interpreted as exploratory and hypothesis generating.

## Conclusion

In this cohort of trauma inpatients, 62.9% experienced moderate to severe pain at the time of assessment despite prescribed analgesia. Paracetamol with tramadol remained the dominant regimen across pain categories, with little visible escalation as pain severity increased. Despite strong recommendations against the use of pethidine as an analgesic, it was frequently prescribed and sometimes combined with another opioid. These findings support review of routine reassessment, clearer escalation pathways and broader multimodal pain-management options within local resource constraints.
